# Implications of human genome structural heterogeneity: functionally related genes tend to reside in organizationally similar genomic regions

**DOI:** 10.1186/1471-2164-15-252

**Published:** 2014-03-31

**Authors:** Arnon Paz, Svetlana Frenkel, Sagi Snir, Valery Kirzhner, Abraham B Korol

**Affiliations:** 1 Department of Evolutionary and Environmental Biology and Institute of Evolution, University of Haifa, Mount Carmel, Haifa 31905, Israel

**Keywords:** Compositional spectra analysis, Sequence organization pattern, Horizontal gene transfer, Whole genome duplication

## Abstract

**Background:**

In an earlier study, we hypothesized that genomic segments with different sequence organization patterns (OPs) might display functional specificity despite their similar GC content. Here we tested this hypothesis by dividing the human genome into 100 kb segments, classifying these segments into five compositional groups according to GC content, and then characterizing each segment within the five groups by oligonucleotide counting (k-mer analysis; also referred to as compositional spectrum analysis, or CSA), to examine the distribution of sequence OPs in the segments. We performed the CSA on the entire DNA, i.e., its coding and non-coding parts the latter being much more abundant in the genome than the former.

**Results:**

We identified 38 OP-type clusters of segments that differ in their compositional spectrum (CS) organization. Many of the segments that shared the same OP type were enriched with genes related to the same biological processes (developmental, signaling, etc.), components of biochemical complexes, or organelles. Thirteen OP-type clusters showed significant enrichment in genes connected to specific gene-ontology terms. Some of these clusters seemed to reflect certain events during periods of horizontal gene transfer and genome expansion, and subsequent evolution of genomic regions requiring coordinated regulation.

**Conclusions:**

There may be a tendency for genes that are involved in the same biological process, complex or organelle to use the same OP, even at a distance of ~ 100 kb from the genes. Although the intergenic DNA is non-coding, the general pattern of sequence organization (e.g., reflected in over-represented oligonucleotide “words”) may be important and were protected, to some extent, in the course of evolution.

## Background

Heterogeneity of the structural characteristics of genomic sequences, such as GC content (isochores), CpG distribution, copy-number variation, and repetitive DNA content has been the subject of numerous studies for decades. Other studies have been devoted to heterogeneity of functional and evolutionary features of the genome, including protein- and non-protein-coding DNA, codon usage, developmental stage- and tissue-specificity of gene expression, distribution of conserved and ultra-conserved regions, recombination and mutation hot and cold spots, and linkage disequilibrium blocks [1-7]. Many genomes have been sequenced and are available for further analysis. Nevertheless, the coding DNA remains the genome’s most investigated component. Analyses of its structure and function have been the basis for a wide variety of studies, ranging from the analysis of functionally related gene groups and gene-alignment-based interspecies comparisons [8] to analysis of gene-adjacent regulatory sequences [9].

The simplest structural characteristic of genomic sequences is their nucleotide composition. Relatively good correspondence of nucleotide composition with the Chargaff’s second parity rule [10] enables disregarding certain differences in the within-strand contents of G vs. C, and A vs. T, and limiting nucleotide-composition studies to an analysis of the molar proportion of G+C in DNA, or GC content. GC content shows high heterogeneity along the genome and correlates with many genomic features, such as recombination rate, abundance of single nucleotide polymorphisms (SNPs), and different types of repetitive elements [11-13]. Of special interest is the well-known correlation of GC content with gene density [12,14]. Furthermore, GC-rich regions contain many compact genes with short introns, whereas genes in GC-poor regions tend to contain larger introns [15]. A correlation between GC content and gene expression has been found as well [11]. It is generally accepted that broadly expressed (housekeeping) genes typically reside in GC-rich regions, although the correlation strength between gene-expression specificity and regional GC content may vary depending on the method used to estimate expression [16]. Furthermore, tissue-specificity of genes varies with their GC content; for example, genes specifically expressed in the central nervous system are more GC rich than housekeeping genes, whereas genes related to germ-line tissues tend to be GC-poor [17,18].

Recent studies have indicated that regulatory sequences of functional gene groups differ in the genome’s GC-poor and GC-rich regions. In addition, sequences that might influence nucleosome positioning and density differ between these two contrasting regions (for review, see [19]). Moreover, different functional gene groups have contrasting base compositions [20], which might explain the relationship between genes’ tissue-specificity and their local GC content. In summary, investigations have shown a correlation between isochore GC content and the resident genes nucleotide composition and functioning.

Analyses of di- and trinucleotide frequencies in five GC-isochore families of the human genome showed unexpected organizational differences between whole isochore sequences, with the corresponding intergenic and coding sequences located in different isochores, in exons and introns [21]. Similar differences were found in gene regulatory regions and in local sequences that might influence nucleosome positioning and density [19]. These differences in the abundance of short oligonucleotides might be related to chromatin organization, which itself plays a role in gene expression and replication timing. An important finding was that genome structural heterogeneity might affect the distribution of gene categories on a larger scale than the classical isochores [22].

In earlier studies, we used the oligonucleotide-counting method (k-mer analysis), referred to as compositional spectrum analysis (CSA) for alignment-free genome comparisons [23-25]. We recently employed this approach in an investigation of organizational heterogeneity of vertebrate genomes with a special focus on the human genome [26]. We considered two types of heterogeneity: compositional (variability of sequence nucleotide frequencies) and organizational (variability of sequence nucleotide orders). A compositional spectrum (CS) comparison of sequences with the same (or similar) GC content can detect groups of genomic segments with very different organizational patterns (OPs). We were interested in testing whether the OP of a genome region affects the type of genes residing there, i.e. whether functionally related genes tend to inhabit regions with similar OPs. To test this, we arbitrarily divided the human genome sequence into 100 kb segments and then classified them into five compositional groups according to their average GC content. For each such GC range, we identified large groups of segments that differed in their CS organization (referred to as OP groups), and compared the genes residing in segments of the same OP group; 13 of the 38 OP groups showed significant enrichment in genes connected to specific gene-ontology (GO) terms. Thus, one of the analyzed groups was considerably enriched in genes connected to the GO terms “mitochondrion” and “ribonucleoprotein complex”. Another OP group was enriched in genes related to a few GO terms: “epithelial cell differentiation”, “epithelium development”, and “keratinocyte differentiation”. These findings enabled us to examine the relationship between gene function and CS organization of the associated genome regions.

## Results

As expected, OP variability could be detected within each compositional group of segments [26]. Correspondingly, segments from each GC range were subdivided into clusters according to OP similarity. Altogether, we identified 38 different OP groups in the five compositional groups. A substantial proportion of the segments contained protein-coding genes (Table 1; see also Additional files 1, 2, 3 and 4 in the supporting material for more details). Most of the genes were located in segments of the L2, H1, and H2 compositional groups with moderate GC content: 37–52% (we employed the names used in the literature for denoting groups with corresponding GC content). As already known and discussed [14,27], there is a strict correlation between GC content and gene density. We found that L2, H1, and H2 compositional groups had higher OP complexity than L1 and H3; therefore, they could be subdivided into more OP clusters. This last trend can be partially explained by simple combinatorial rules based on A-C-T-G nucleotide distribution [26]. We found that each OP group contained segments that were widely spread among and within different chromosomes. It is worth noting that our CSA addressed the whole segment sequence, including both the gene sequences and intergenic DNA. (see Additional file 1, 2, 3, and 4).

**Table 1:** Variability of organizational patterns within the five GC-range groups

**GC range**	**OP groups**	**Segments**	**Protein coding genes**	**Genes per segment**
L1, <37%	5	5350	641	0.12
L2, 37-42%,	9	10610	4131	0.39
H1, 42-47%	15	8738	6701	0.77
H2, 47-52%	7	3269	4727	1.44
H3, >52%	2	901	2059	2.29
Total	38	28868	18259	0.63

### Different OP groups are enriched in different functional gene categories

**Figure 1:**
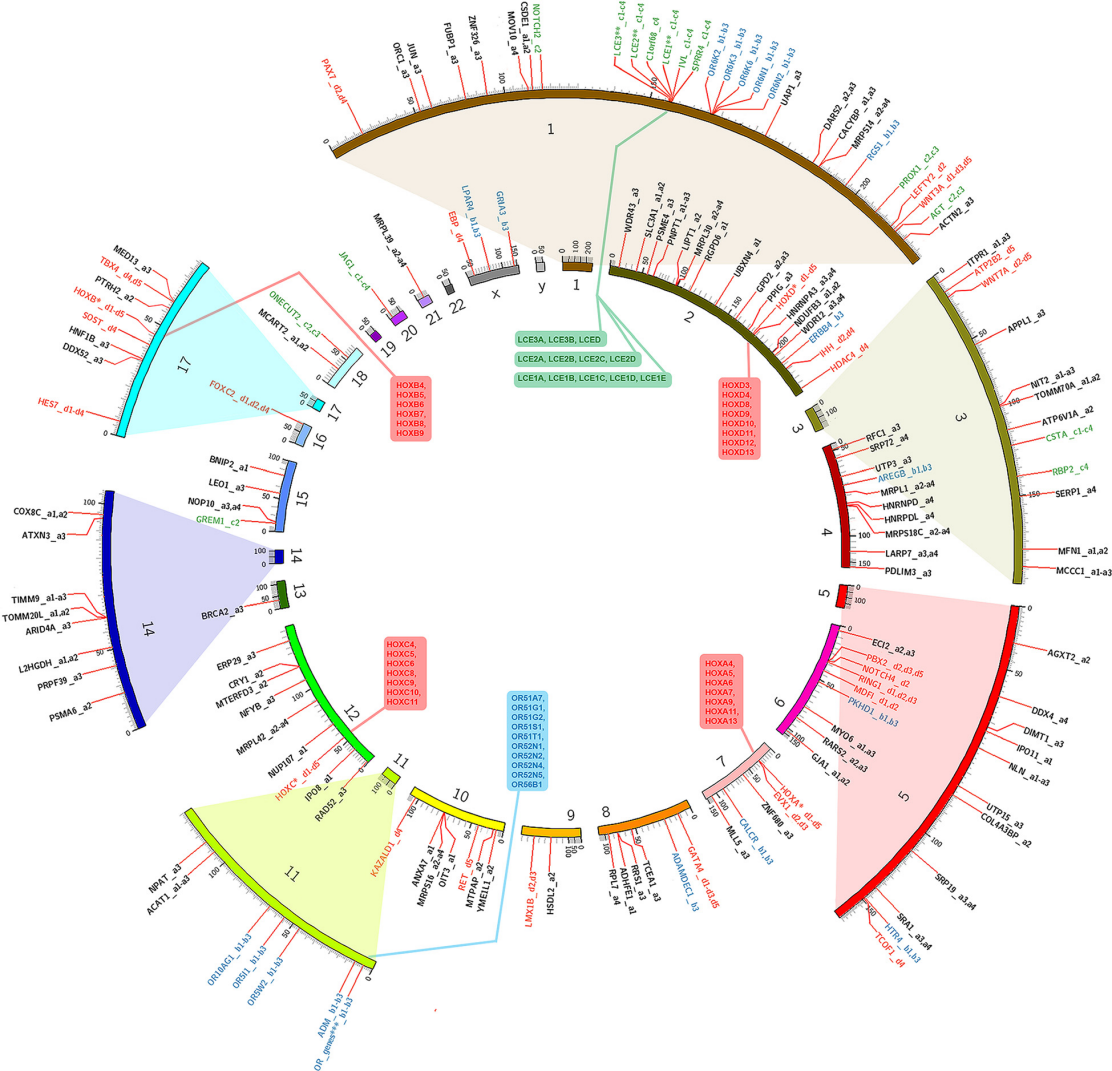
**Genes provided the GO enrichment of four organizational pattern clusters, which showed the most significant GO enrichments. L2-a cluster (**94 out of 392 genes associated with the enriched GO terms**) **marked by black labels; **L2-h cluster** 29 out of 126 genes marked by blue labels; **H1-i cluster** 24 out of 326 genes marked by green labels; **H2-a** cluster 50 out of 606 genes marked by red labels. Note that different chromosomal regions are shown in the figure in varying scales in order to enable accurate indication of corresponding gene(s) residence. List of enriched GO terms (Benjamini p-values of the GO term enrichments are shown in brackets): (**a1)** organelle envelope (0.001174); **(a2)** mitochondrion (0.000760); **(a3)** membrane-enclosed lumen (0.002300); **(a4)** ribonucleoprotein complex (0.002055); **(b1)** G-protein coupled receptor protein signaling pathway (0.002585); **(b2)** sensory perception of smell (0.003231); **(b3)** cell surface receptor linked signal transduction (0.033179); **(c1)** keratinocyte differentiation (4.07 × 10^−9^); **(c2)** epithelium development (6.78 × 10^−7^); **(c3)** epithelial cell differentiation (2.83 × 10^−9^); **(c4)** ectoderm development (4.55 × 10^−5^); **(d1)** anterior/posterior pattern formation (1.9 × 10^−10^); **(d2)** pattern specification process (2.0 × 10^−10^); **(d3)** regionalization (1.9 × 10^−10^); **(d4)** skeletal system development (9.7 × 10^−10^); **(d5)** embryonic morphogenesis (0.000293).

Previous studies have clearly demonstrated that compositionally different genomic segments (GC-rich vs. GC-poor) may also differ with respect to their genes’ functional categories. Thus, a considerably higher proportion of housekeeping genes was found in GC-rich vs. GC-poor human genome regions [17]. We hypothesized that genomic segments with different OPs would display functional specificity despite GC-content similarity [26]. To test this, CSA was applied to 100 kb segments in the five compositional groups with GC ranges corresponding to the classical isochores. We found that segments sharing the same OP type are significantly enriched in genes related to the same biological process (developmental, signaling, etc.), biochemical complex, or organellar components (although this did not exclude enrichment with genes connected to other categories). These relations were identified by screening 38 OP-type clusters: one-third showed significant enrichment for genes connected to specific GO terms. The main findings are illustrated by several examples from the compositional groups L2, H1, and H2 presented in Figure 1, and more comprehensive results are shown in Additional files 1, 2, 3 and 4. We also tested genome organization patterns using CSA based on the two-letter alphabet (purines, R = G or A and pyrimidines, Y = C or T). In this CSA version, the abundance of 20-mer words was counted and compared to that of 10-mer words in the full A, T, G, C alphabet [24,25]. Although the results were not identical, they are similar and highly correlated.

### Repeat-masking test

Bearing in mind that repetitive DNA sequences comprise a considerable proportion of the human genome, we checked for an influence of these repeats on OP group size and GO-term enrichment by employing the RepeatMasker tool (Smit, AFA, Hubley, R & Green, P. RepeatMasker Open-3.0 1996–2010 unpublished data <http://www.repeatmasker.org>) on segments with GC = 42–47%. In addition, we conducted OP-group identification within the repeat-masked (RM) sequence, and analyzed the enrichment of the resulting OPs by genes related to the same GO terms. Differences in oligonucleotide frequencies between OP groups might come from repeat sequences; therefore, the two tests cannot be expected to generate identical patterns. Nonetheless, the results in the RM test seemed quite similar to those in the original test. In the original test, we found that 5 of the 15 OP groups from the GC = 42–47% range were enriched for 30 GO terms (connected to 186 genes); for RM sequences, 6 out of 16 OP groups were enriched for 46 GO terms (connected to 291 genes). Two out of the five OP groups that were found to be significantly enriched for specific GO terms in the original test fully coincided with those in the RM test. In total, 2/3 of the GO terms enriched in certain OP groups in the original test were also enriched in certain OP groups under the RM test. We believe that some of the repeat sequences, like other inter-genic DNA (e.g., the “genome dark matter” [1-3]), might be important for regulation of gene expression or other processes in which DNA sequences of nearby genes are involved (replication, DNA repair, etc.). Therefore, we decided that it would make more sense to show the CSA results obtained for the whole DNA, including the repeats.

### Enrichment of OP cluster from L2 group for the GO term “mitochondrion”

A subgroup corresponding to the L2 compositional group with similar OP was termed as **L2-a** cluster (**L2-a** OP group). Out of 392 genes harbored by this group, 39 were related to the GO term “mitochondrion” (Benjamini p-value: 7.67 × 10^−4^) and are distributed among 34 loci of 15 chromosomes (see Figure 1). More than 900 chromosomal genes encoding human proteins are considered to be targeted to the mitochondria [28]; this number may be even larger [29]. Many of the chromosomal genes encoding for these mitochondrion-targeted proteins might have originated from the ancestral alpha-proteobacteria symbiont genome (the suggested mitochondrion progenitor) and been transferred to eukaryotic chromosomes by horizontal gene transfer (HGT) [30,31]. It is now widely accepted that genes related not only to metabolism, but also to producing the eukaryotic membranes and nucleus were transferred from the endosymbiont [32,33].

The GO term “intracellular non-membrane-bounded organelle” was also significantly enriched in the **L2-a** OP cluster (connected to 70 genes; Benjamini p-value: 0.00771). The GO term “nuclear envelope” was connected to 11 genes within the **L2-a** cluster, albeit with a higher Benjamini p-value (0.0522) indicating that among these 11 genes, no more than one might be considered a false positive (see Additional file 2). Twenty-one genes in the **L2-a** OP cluster were found to be connected to the GO term “ribonucleoprotein complex” (Benjamini p-value: 0.00206) and were located in 10 chromosomes (19 loci). Some of these genes’ products are indeed mitochondrial ribosomal proteins: MRPS14, MRPS16, MRPS18C, MRPL42, MRPL1, MRPL39, and MRPL30. However, the group also includes non-mitochondrial genes, for example, *SRA1*. Transcripts of *SRA1* have both coding and non-coding co-activator activities; along with *SRA1* protein product SRAP, it performs mixed co-activator/repressor functions in differentiation and metabolism. Other examples are *DDX4*, which functions in germ-cell development, and *WDR12*, a WD40 repeat protein that is crucial for processing 32S precursor ribosomal RNA (rRNA) and for cell proliferation. Also worth noting is that in the L2 group, RY-based CS analysis revealed an OP cluster very similar to **L2-a** with respect to GO-term enrichment; 9 of the 14 GO terms that were found to be enriched in this RY OP cluster coincided with the terms enriched in the **L2-a** OP cluster obtained using k-mer CSA in the standard four-letter alphabet (see Additional file 2).

### OP cluster from L2 group enriched in G-protein-coupled receptors (GPCRs)

The cluster termed **L2-h** included 126 genes. Twenty eight of these genes, which were connected to GO term “G-protein-coupled receptor protein signaling pathway” (Benjamini p-value: 0.0026), were located in 11 loci on seven chromosomes (see Figure 1). Moreover, 19 of these genes were also connected to GO term “sensory perception of smell” (Benjamini p-value: 0.00323) and encoded olfactory receptors (ORs). The OR subgroup of GPCRs is one of the largest mammalian genome superfamilies. In the human genome, this group includes approximately 960 genes, although ~51% of them are actually pseudogenes [34]. Each OR gene is approximately 1 kb in length, intronless, and found in clusters on almost all chromosomes. In addition, like all GPCRs, each OR gene shares a common molecular architecture consisting of seven transmembrane domains.

### H1 cluster related to epithelial cell differentiation

The OP cluster **H1-i** included 326 genes, 21 of which were connected to the GO term “epithelial cell differentiation” (Benjamini p-value: 2.83 × 10^−9^); this group and four additional genes were connected to the GO term “epithelium development” (Benjamini p-value: 6.78 × 10^−7^). Although the 25 genes were located in eight loci of five different chromosomes, 18 were clustered on chromosome 1q, including 16 late cornified envelope (LCE) genes that were also connected with the GO term “keratinocyte differentiation” (Benjamini p-value: 4.07 × 10^−9^). Cluster **H1-i** also included genes that have additional roles in the epithelium as well as in differentiation and maturation of other tissues (see Figure 1, Additional file 3). Some examples of these include: *SPRR4*, induced by ultraviolet light and other environmental stresses [35]; *NOTCH2*, known to delay hepatoblast maturation during early hepatic organogenesis, and *JAG1* playing a role in hematopoiesis [36]; *GREM1*, which is involved in regulating organogenesis, body patterning, and tissue differentiation [37]; *ONECUT1,* which encodes a transcription factor mediating complex processes in the liver and pancreas related to cell proliferation, cell-cycle regulation, cell differentiation, and organogenesis [38], and *AGT*, reported to be involved in the epithelial-to-mesenchymal transition in renal epithelial cells [39] and in maintaining blood pressure [40] (see Additional file 3).

### A developmental OP cluster in the H2 group

One of the clusters from the H2 segment group (termed **H2-a**) included 606 genes, 40 of which proved to be connected to the GO term “skeletal system development” (Benjamini p-value: 9.7 × 10^−10^). These genes were located in 18 loci on 12 chromosomes, with the largest proportion represented by *HOX* genes (see Figure 1, Additional file 4). *HOX* proteins are transcription factors (TFs) with a 60-amino-acid-long DNA-binding homeodomain. They can function as enhancers or repressors, and many of them participate in morphological or developmental pattern regulation. *HOX* genes are located in four chromosomal loci (2q31, 7p15, 12q13, and 17q21), originating from duplication of a single ancestral cluster [41,42]. The **H2-a** OP cluster included additional developmental genes: *PAX7*, a TF gene with a paired-type homeodomain that plays a critical role during fetal development; *RING1*, which encodes a TF associated with the multimeric polycomb protein complex; *WNT3A* and *WNT7A* implicated in oncogenesis and in several developmental processes, including cell-fate regulation and patterning during embryogenesis; *LEFT2* which plays a role in organ system developmental left–right asymmetry determination; *MMP9*, encoding a matrix metalloproteinase involved in embryonic development, reproduction, and tissue remodeling; *NOTCH4,* involved in developmental processes by controlling cell-fate decisions and interaction between physically adjacent cells; *RET*, playing a crucial role in neural crest development; *HES7*, a TF implicated in correct axial skeleton patterning; and *HDAC4*, encoding histone deacetylase which represses transcription when tethered to a promoter. As with the **L2-a** OP cluster, 13 of the 17 GO terms found to be enriched in the RY OP cluster coincided with 13 of the 24 GO terms enriched in the **H2-a** OP cluster, obtained using the standard four-letter alphabet (see Additional file 4).

### Additional interesting cases of GO-term enrichment in specific OP clusters

The following are further interesting examples of functionally significant OP clusters from the L2 and H1 compositional segment groups. More details, including the chromosomal loci and the names of included genes, can be found in Additional files 1, 2, 3 and 4.

(i) **L2-b** and **H1-m** clusters enriched for GO terms “keratin filaments” (20 genes; Benjamini p-value: 0.00210), and “intermediate filament” (19 genes, mostly keratins; Benjamini p-value: 8.40 × 10^−9^), respectively. This example demonstrates that functionally similar OP clusters can be found in diverse GC groups, implying that in such cases, OP rather than GC content plays a role in the positioning of the corresponding genes.

(ii) **L2-f** cluster enriched for GO term “keratin-associated proteins” (8 genes; Benjamini p-value: 2.89 × 10^−9^).

(iii) **L2-e** and **L2-g** clusters enriched for GO terms “G-protein-coupled olfactory receptor, class II” (21 genes; Benjamini p-value: 0.0237), and “G-protein-coupled receptor protein signaling pathway” (63 genes; Benjamini p-value: 0.0357), respectively.

(iv) **H1-e** and **H1-j** OP clusters enriched for GO terms “nucleosome” (27 genes, mostly histones; Benjamini p-value: 0.00150), and “Kegg pathway: Systematic lupus erythmatosus” (15 genes, mostly histones; Benjamini p-value: 0.0354), respectively.

(v) **H1-h** cluster enriched for GO term “homophilic cell adhesion” (9 genes, all protocadherines; Benjamini p-value: 4.68 × 10^−6^).

### Non-randomness of enrichments

The aforementioned results prompted the question of whether similar function-related detection levels would be obtained if groups of the same size as the original OP groups were built by taking the segments at random within the same GC limits. This question was addressed in the following way. Within each of the three main GC ranges (L2: 37–42%, H1: 42–47%, and H2: 47–52%), we randomly distributed the 100-kb segments into groups with the same sizes as the 31 OP groups obtained by CS comparison. This was repeated 10 times and the resultant 310 random groups of segments were checked for enrichment in genes related to specific GO terms. Such enrichments were found in 65 of the 310 groups, which is significantly less than the corresponding proportions in real OP groups, where 13 out the 31 groups were enriched by functionally related genes (p-value = 0.0106 by the Fisher two-tailed exact test). We further compared the OP groups and random groups with respect to obtained enrichment significance by GO term (Table 2), by applying the Mann–Whitney U test [43] to compare the distribution of -log_10_ (p-value) scores in OP groups and in random groups. Enrichment in the OP groups was characterized by considerably higher -log_10_ (p-value) scores than in the randomly obtained groups (adjusted Z = 2.719, P = 0.0065).

**Table 2:** Average characteristics of OP and random groups and results of their comparison using Mann–Whitney U test

**Group characteristics**	**Mean ± SE**		**Z**	**P**
	**OP groups**	**Random groups**		
-log_10_(p-value) [Benjamini]	3.15 ± 0.25	2.27 ± 0.09	2.719	0.0065
Number of GO terms	7.11 ± 1.81	4.14 ± 0.62	2.502	0.0124
No of segments with GO connected genes	46.6 ± 16.3	16.8 ± 4.3	2.565	0.0103
Ratio of involved segments/all segments in the cluster	0.071 ± 0.021	0.032 ± 0.012	2.923	0.0035

We also analyzed three additional characteristics of the OP and random groups, which were found to be enriched in at least one group of functionally related genes. These included: (i) the number of GO terms per “non-empty” OP group or its random analogue with the assumption that GO-term enrichment for real OP groups should be higher than for randomly formed groups; (ii) the number of segments containing functionally related genes connected with a GO term(s); here we assumed that enrichment due to high numbers of such “involved” segments reflects a positive correlation between the genes’ shared functional relevance and their shared residence in genome-wide distributed segments with similar sequence organization; in random clusters on the other hand, such correlations could arise only due to closely linked (within 100 kb) genes; (iii) the ratio of the involved segments to all segments in the cluster (a normalized variant of criterion **ii**). For all three criteria, the randomly combined groups showed significantly lower enrichment compared to the real OP groups formed from 100 kb segments using k-mer analysis to assess sequence similarity (see Table 2).

## Discussion

GC-content variation within the genome displayed in the form of isochores [14,44], and its function in human and other vertebrate genomes have been targeted in many studies. In this analysis, we focused on another aspect of genome organization: possible functional correlates of human genome “organizational heterogeneity” [26], displayed in the variation in the abundances of different oligonucleotide “words” (k-mers). In particular, we were interested in testing whether region-specific “word” usage (regional variation of the genome “accent” [25]) may have functional or evolutionary implications, or both. We used CS analysis [24] to identify clusters of 100-kb segments with similar OPs within five major GC groups (with the same GC-content as in the main isochore groups). We further looked for GO-term enrichment in each of the OP clusters. We revealed that, in many cases, OP clusters were significantly enriched in genes involved in the same biological process (developmental, signaling, etc.). There were also cases of genes within OP clusters involved in the same biochemical complex, or organelle. Moreover, organizational similarity of the clustered 100-kb segments and functional similarity of genes belonging to these segments were observed despite the dispersed genome-segment locations. As the calculations were made on the whole chromosomal sequence, the underlying OPs should include both coding and non-coding DNA (introns and inter-genic DNA), the latter being much more abundant in the genome than the former (with a relative proportion of approximately 20:1). Therefore, we concluded that there might be a tendency for genes involved in the same biological process/complex/organelle to use the same OP, even far away–up to 100 kb–from the genes. We do not know the mechanism underlying this similarity, but these sites might share preferred DNA-repair mechanisms with a resulting similar bias for specific use of nucleotides and oligonucleotide “words” [25]. Another possibility is that genes of the same OP cluster are controlled by similar regulatory sequences (see also Discussion in [26]). In this case, although the intergenic DNA is non-coding, the general sequence organization (the over-represented oligonucleotide words, or the “accent” [25]) may be very important and should be conserved.

The complexity of the human genome involves many layers of large-scale duplications (segmental, chromosomal, and whole genome) and expansion of the gene repertoire of the predecessor genomes by HGT. Some of our OP clusters might indeed reflect certain events during HGT and genome-expansion periods, and the subsequent evolution of genomic regions requiring coordinated regulation: **(a)** HGT from the alpha-proteobacteria symbiont (mitochondria) to the primitive eukaryotes [30,31] that may, to some extent, be related to the L2-a OP cluster; **(b)** the emergence of vertebrates from chordates by the two-round (2R) whole genome duplication (WGD) [45-47] relevant to the H2-a OP-type cluster; **(c)** the segmental duplications that enabled adaptation of vertebrates to life out of water, including expansion of olfactory receptor genes [48-50] relevant to the L2-h OP cluster; and **(d)** expansion of gene families related to the evolution of mammalian skin (H1-i OP cluster) that is better adapted to water homeostasis than that of amphibians [51,52]. Regarding the last statement, aside from the LCE genes, GO terms related to keratins were found to be significantly enriched in some other OP clusters (L2-h, L2-f and H1-m; see section “Additional interesting cases” in the Results, and Additional files 2, 3 and 4). In the following, we discuss the possible evolutionary meaning of the corresponding findings.

### Evolution of genome regions harboring “mitochondrion-related” genes

The deepest genome expansion layer potentially reflected in the CSA results is the mitochondrion-related gene enrichment in the **L2-a** OP cluster. HGT from the alpha-proteobacteria symbiont to its eukaryotic host-cell genome happened 2 BYA. Some of the complexities and the mixed-up eukaryote genome might have resulted from the fact that bacteria that gave rise to mitochondria did not shrivel up into ATP-producing factories. Instead, many of their genes were transferred to their hosts’ genomes [30-33] and, therefore, might subsequently have participated in producing the eukaryotic membranes and nucleus [32,33]. In addition to the coding-gene transfers that benefitted the host, there were probably many cases of DNA sequence insertion that could not be expressed (if the insertion was to chromosomal sites lacking the correct regulation signals for the host transcription machinery).

A preferential insertion of large, mitochondrial-origin DNA fragments (average insertion size 1.3 ± 0.73 Mb) to pericentromeric and subtelomeric regions of human chromosomes was recently suggested by Moon et al. [53]. However, as about half of the mitochondrion-related genes and pseudogenes are not clustered (they are spread over different chromosomes) [54], it seems that these genes were rearranged during the subsequent genome evolution by non-homologous end-joining repair of DNA double-strand breaks [53]. Our CS analysis was performed on 100-kb-length segments; hence both genes and intergenic DNA might be included on the same segment. Therefore, with regard to mitochondrial-origin genes, the fragments transferred from the symbiont (genes and intergenic DNA) might have conserved their OP even further away from the genes themselves, at least within the range of 100 kb. Alternatively, and more likely, the extant gene locations do not directly represent the sites of ancient HGT events, but are secondary, resulting from rearrangements and biased reinsertions into the host chromosomal sites that share a similar OP. According to our results, the OP seems to be conserved in both scenarios (direct HGT or rearrangement).

If our interpretation is correct, then analyzing OP structure of additional eukaryotic genomes should reveal enrichment for the GO term “mitochondrion” in at least one OP type. However, such an analysis is beyond the scope of the reported investigation.

### OPs and WGD

The refined 2R hypothesis assumes that two rounds of WGD took place after the emergence of urochordates and before the radiation of jawed vertebrates, some 550 MYA [45-47,55,56]. In almost all of the debates surrounding the 2R hypothesis, the *HOX* gene family which follows a *4:1 rule* in the number of vertebrate to invertebrate genes is used by proponents as a supporting argument [46,47,57]. The duplication of genomic loci provided increased flexibility, allowing the generation of new expression patterns, and was critical for the emergence of morphological novelties [58,59]. This “regulatory evolution” of the four *HOX* gene clusters involved enhancer elements distant from their target promoters [47,60,61], where potential interference with modules of ancestral control was minimized [47]. We assume that the CSA results on *HOX* gene enrichment in the **H2-a** OP cluster reflect the aforementioned large-scale genomic expansion event and that OP conservation at a distance of ≤100 kb from the gene edges following these ancient duplication events is an important tier of process regulation.

#### OP and segmental duplications in the vertebrates’ “out of water” evolution

Expansion of several gene families occurred during the emergence of the class Amphibia, in the Devonian Period, about 375 MYA. This might be represented by the “signals” from cluster **L2-h** with its significant olfactory receptor (OR) enrichment. It has been suggested that expansion of the OR gene family was positively selected for in amphibians evolving from the fish lineage during adaptation to terrestrial environments. This assumption is based on variation in OR gene number: about 150 in zebrafish and 15 in pufferfish [50] as compared to 665 in *Xenopus tropicalis* and 700–1700 in terrestrial mammals [62,63]. Most of the *X. tropicalis* and mammalian OR genes are class II genes [62] that might be specialized for detecting airborne odorants [49], which outnumber waterborne odorants.

The duplication of skin-related LCE genes (OP cluster **H1-i**) seems to have occurred during the emergence of the class Mammalia about 200–120 MYA. The LCE gene cluster on 1q21 is located within a 2-Mb region called ‘the epidermal differentiation complex’ which also includes additional clusters of gene families encoding major proteins of late epidermal differentiation (S100 and S100-fused type proteins, involucrin, loricrin, and the SPRRs (small proline-rich proteins) - a sister protein family of LCE [52]). Such protein family-clustered organization suggests duplication in ancestors adapting to changes in terrestrial conditions in the course of evolution [51,52]. It has been suggested that in mammals, the developed “grouping” of SPRRs is better adjusted to subtle cellular and environmental stimuli than a single or a few genes, as clusters could constitute an “extended promoter” [51]. This might also be true for the LCE genes responding “group-wise” to environmental stimuli, such as calcium levels and ultraviolet light, though only *LCE3B* and *LCE3C* encode proteins involved in barrier repair after injury or inflammation [64].

Similar to the afore-discussed major examples of OP-function correlations, the examples provided in the section “Additional interesting cases”, may also reflect important events in vertebrate evolution. Thus, cases **(i)** and **(ii)** (see Results) might relate to expansion events of protein/gene families related to keratin, the key structural material making up the outer layer of skin and hair. The expansion of keratin families occurred during the emergence of the Amniotes (340–306 MYA) and played essential roles in the formation of rigid and resistant hair shafts [65]. Case **(iii)** is an additional example of the expansion of ORs and other GPCRs. The example in case (**iv**) shows that histone genes from different chromosomal loci belong to the same OP clusters. Gene duplication has prevailed as the major mechanism in providing the eukaryotic cell with the required histone number and diversity [66]. Histone variability in multicellular organisms is required to accommodate the different packing needs and gene-expression regulation in different cell types and developmental stages.

## Conclusions

In many examples, the similarity of the local microgeographical “accents”, that is, gene-harboring segments belonging to the same OP cluster, seems to have derived from duplications of one origin. This might be a large-scale duplication, for example, WGD presumably reflected by the **H2-a** OP cluster, or segmental duplications presumably exemplified by the **L2-h** and **H1-i** clusters. Duplications of minor-sized chromosomal DNA stretches are more abundant and could have occurred due to unequal crossing-over between misaligned homologous chromosomes. Regardless of the exact scenario for the duplication event, the results point to conservation of DNA sequence OPs that are distant (10–100 kb) from the genes’ edges. These sequences might be important in regulation of gene expression, or in other processes in which DNA sequences of nearby genes are involved (replication, DNA repair, etc.). Our results presume that many genes belonging to the same OP cluster do not share a common origin location. As we do not know the mechanisms for the preferred use of oligonucleotide “words”, we can only suggest that some of these genes belong to the same “transcription factories” and, as such, may also share a DNA-repair mechanism, such as transcription-coupled repair. If transcription of these genes occurs at the same time, they might share the same regulation signals: short, moderate, or even long oligonucleotide “words” at a distance from the gene edges. Similar to a recent suggestion [67] that eukaryotic species’ evolutionary transitions are associated with codon bias in genes encoding functionally related proteins, we suggest that not only the coding sequences, but also sequences at a distance from the duplicated genes (e.g., ~100 kb) might share the same “accent”: there could be a bias in the repertoire of oligonucleotide “words” that might have been conserved in many cases during the course of eukaryote evolution.

## Methods

### Calculating compositional spectra

Consider a set *W*, including *n* different words (oligonucleotides), *w*_*i*_, with length *L* from the standard DNA alphabet {A, C, T, G}. For any word *w*_*i*_ from set *W* and a chosen sequence *S*, the observed number of matches *m* = *m* (*w*_*i*_) can be calculated with a certain number of allowed replacements (*r*), for example, 0, 1, or 2 (*r* = 2). Let *M* = ∑_*i*_*m*_*i*_. We refer to the frequency distribution *F*(*W*, *S*) based on frequencies *f*_*i*_ = *m*_*i*_/*M* as the compositional spectrum (CS) of sequence S relative to the set of words *W*. For every set of words *W*, it is possible to produce a set of complementary words *W*′, where the word *w*′_*n*_ is the complementary reverse of word *w*_*n*_[26].

### Calculating CS distances between DNA sequences

We define the difference *d* between two sequences, *S*_1_ and *S*_2,_ as the distance between their spectra *F*(*W*, *S*_1_) and *F*(*W*, *S*_2_). We use the Spearman rank correlation coefficient *r*_s_ between two CSs computed on the compared sequences as a base for calculating distance *d*. The inter- or intra-genomic similarities and dissimilarities can be displayed by distance matrices of pairwise CS comparisons. The distance matrices can be used to select similar segments and their clustering; for example, by using the neighbor-joining algorithm.

### Detection of OP groups of segments

To analyze the relationship between genes located in regions with the same OP, we classified all 100-kb segments into five groups according to their average GC content. We did not use the division of sequences into known isochore families [68,69], but we did use the same “borderline” GC content values for separating and designating the obtained GC groups by the isochore family names: L1: GC content < 37%, L2: 37–42%, H1: 42–47%, H2: 47–52%, and H3: GC content >52%. We conducted neighbor-joining clustering based on between-segment dissimilarities within each GC range to obtain groups of segments with relatively similar OPs. For each OP group, we detected all genes residing in the corresponding segments, according to their start and end positions obtained from the Ensembl genome browser (http://www.ensembl.org/). To identify the OP groups enriched in genes involved in the same biological process, we compared every gene list with all genes located in the corresponding GC group using DAVID (Database for Annotation, Visualization and Integrated Discovery [70]). To account for multiple testing, Benjamini corrected p-values [71] were employed for the obtained enrichments.

## Abbreviations

CSA: Compositional spectra analysis; OP: Organization pattern; SNP: Single nucleotide polymorphism; GO: Gene ontology; WGD: Whole genome duplication.

## Competing interests

The authors declare that they have no competing interests.

## Authors’ contributions

AP participated in the analysis and interpretation of the results and drafted the manuscript. SF participated in designing the analysis tools, performed the k-mer analysis, and participated in writing the manuscript. SS participated in the analysis and discussion of obtained results. VK conceived and designed the analytical tools. AK conceived the study, designed and coordinated the analysis, and participated in writing the manuscript. All authors read and approved the final manuscript.

## Authors’ information

Arnon Paz and Svetlana Frenkel shared first co-authorship.

## Supplementary Material

Additional file 1 An example of table, presenting GO terms, proved to be significantly enriched in corresponding OP group, Benjamini p-values of this enrichments and genes, related to each GO term. Click here for file

Additional file 2 The Organizational Pattern (OP) groups, GO terms enriched in these groups with corresponding Benjamini p-values of the GO term enrichments, and genes providing this enrichment for “L2” GC group. Click here for file

Additional file 3 The Organizational Pattern (OP) groups, GO terms enriched in these groups with corresponding Benjamini p-values of the GO term enrichments, and genes providing this enrichment for “H1” GC group. Click here for file

Additional file 4 The Organizational Pattern (OP) groups, GO terms enriched in these groups with corresponding Benjamini p-values of the GO term enrichments, and genes providing this enrichment for “H2” GC group. Click here for file

## References

[B1] Karlin S Ladunga I Blaisdell BE Heterogeneity of genomes: measures and values Proc Natl Acad Sci U S A 1994 91 12837 12841 10.1073/pnas.91.26.12837 7809131 PMC45535

[B2] Lercher MJ Urrutia AO Pavlícek A Hurst LD A unification of mosaic structures in the human genome Hum Mol Gen 2003 12 2411 2510 doi:1093/hmg/ddg251 10.1093/hmg/ddg251 12915446

[B3] Weir BS Cardon LR Anderson AD Nielsen DM Hill WG Measures of human population structure show heterogeneity among genomic regions Genome Res 2005 15 1468 1476 doi:10.1101/gr.4398405 10.1101/gr.4398405 16251456 PMC1310634

[B4] Sellis D Provata A Almirantis Y Alu and LINE1 distributions in the human chromosomes: evidence of global genomic organization expressed in the form of power laws Mol Biol Evol 2007 24 2385 2399 doi:10.1093/molbev/msm181 10.1093/molbev/msm181 17728280

[B5] Schmegner C Hameister H Vogel W Assum G Isochores and replication time zones: a perfect match Cytogenet Genome Res 2007 116 167 172 doi:10.1159/000098182 10.1159/000098182 17317955

[B6] Eory L Halligan DL Keightley PD Distributions of selectively constrained sites and deleterious mutation rates in the hominid and murid genomes Mol Biol Evol 2010 27 177 192 doi:10.1093/molbev/msp219 10.1093/molbev/msp219 19759235

[B7] Pink CJ Hurst LD Late replicating domains are highly recombining in females but have low male recombination rates: implications for isochore evolution PLoS One 2011 6 e24480 doi:10.1371/journal.pone.0024480 10.1371/journal.pone.0024480 21949720 PMC3176772

[B8] Chen Z Ye H Zhou L Cheng C-HC Chen L A gene family-based method for interspecies comparisons of sequencing-based transcriptomes and its use in environmental adaptation analysis J Genet Genomics 2010 37 205 218 doi:10.1016/S1673-8527(09)60039-4 10.1016/S1673-8527(09)60039-4 20347830

[B9] Jaksik R Rzeszowska-Wolny J The distribution of GC nucleotides and regulatory sequence motifs in genes and their adjacent sequences Gene 2012 492 375 381 doi:10.1016/j.gene.2011.10.050 10.1016/j.gene.2011.10.050 22101187

[B10] Bell SJ Forsdyke DR Deviations from Chargaff’s second parity rule correlate with direction of transcription J Theor Biol 1999 197 63 76 doi:10.1006/jtbi.1998.0858 10.1006/jtbi.1998.0858 10036208

[B11] Versteeg R van Schaik BDC Van Batenburg MF Roos M Monajemi R Caron H Bussemaker HJ van Kampen AHC The human transcriptome map reveals extremes in gene density, intron length, GC content, and repeat pattern for domains of highly and weakly expressed genes Genome Res 2003 13 1998 2004 doi:10.1101/gr.1649303 10.1101/gr.1649303 12915492 PMC403669

[B12] Freudenberg J Wang M Yang Y Li W Partial correlation analysis indicates causal relationships between GC-content, exon density and recombination rate in the human genome BMC Bioinformatics 2009 10 Suppl 1 S66 doi:10.1186/1471-2105-10-S1-S66 10.1186/1471-2105-10-S1-S66 19208170 PMC2648766

[B13] Pozzoli U Menozzi G Fumagalli M Cereda M Comi GP Cagliani R Bresolin N Sironi M Both selective and neutral processes drive GC content evolution in the human genome BMC Evol Biol 2008 8 99 doi:10.1186/1471-2148-8-99 10.1186/1471-2148-8-99 18371205 PMC2292697

[B14] Costantini M Cammarano R Bernardi G The evolution of isochore patterns in vertebrate genomes BMC Genomics 2009 10 146 doi:10.1186/1471-2164-10-146 10.1186/1471-2164-10-146 19344507 PMC2678159

[B15] Lander ES Linton LM Birren B Nusbaum C Zody MC Baldwin J Devon K Dewar K Doyle M FitzHugh W Funke R Gage D Harris K Heaford A Howland J Kann L Lehoczky J LeVine R McEwan P McKernan K Meldrim J Mesirov JP Miranda C Morris W Naylor J Raymond C Rosetti M Santos R Sheridan A Sougnez C Initial sequencing and analysis of the human genome Nature 2001 409 860 921 doi:10.1038/35057062 10.1038/35057062 11237011

[B16] Sémon M Mouchiroud D Duret L Relationship between gene expression and GC-content in mammals: statistical significance and biological relevance Hum Mol Gen 2005 14 421 427 doi:10.1093/hmg/ddi038 15590696 10.1093/hmg/ddi038

[B17] Vinogradov AE Isochores and tissue-specificity Nucleic Acids Res 2003 31 5212 5220 doi:10.1093/nar/gkg699 10.1093/nar/gkg699 12930973 PMC212799

[B18] Vinogradov AE Noncoding DNA, isochores and gene expression: nucleosome formation potential Nucleic Acids Res 2005 33 559 563 doi:10.1093/nar/gki184 10.1093/nar/gki184 15673716 PMC548339

[B19] Arhondakis S Auletta F Bernardi G Isochores and the regulation of gene expression in the human genome Genome Biol Evol 2011 3 1080 1089 doi:10.1093/gbe/evr017 10.1093/gbe/evr017 21979159 PMC3227402

[B20] D’Onofrio G Ghosh TC Saccone S Different functional classes of genes are characterized by different compositional properties FEBS Lett 2007 581 5819 5824 doi:10.1016/j.febslet.2007.11.052 10.1016/j.febslet.2007.11.052 18037382

[B21] Costantini M Bernardi G The short-sequence designs of isochores from the human genome Proc Natl Acad Sci U S A 2008 105 13971 13976 doi:10.1073/pnas.0803916105 10.1073/pnas.0803916105 18780784 PMC2532971

[B22] Carpena P Oliver JL Hackenberg M Coronado AV Barturen G Bernaola-Galván P High-level organization of isochores into gigantic superstructures in the human genome Phys Rev E 2011 83 031908 doi:10.1103/PhysRevE.83.031908 10.1103/PhysRevE.83.031908 21517526

[B23] Kirzhner V Korol A Bolshoy A Nevo E Compositional spectrum—revealing patterns for genomic sequence characterization and comparison Phys A Stat Mech its Appl 2002 312 447 457 doi:10.1016/S0378-4371(02)00843-9 10.1016/S0378-4371(02)00843-9

[B24] Kirzhner V Paz A Volkovich Z Nevo E Korol A Different clustering of genomes across life using the A-T-C-G and degenerate R-Y alphabets: early and late signaling on genome evolution? J Mol Evol 2007 64 448 456 doi:10.1007/s00239-006-0178-8 10.1007/s00239-006-0178-8 17479343

[B25] Paz A Kirzhner V Nevo E Korol A Coevolution of DNA-interacting proteins and genome “dialect” Mol Biol Evol 2006 23 56 64 doi:10.1093/molbev/msj007 16151189 10.1093/molbev/msj007

[B26] Frenkel S Kirzhner V Korol A Organizational heterogeneity of vertebrate genomes PLoS One 2012 7 e32076 doi:10.1371/journal.pone.0032076 10.1371/journal.pone.0032076 22384143 PMC3288070

[B27] Saccone S Federico C Solovei I Croquette MF Della Valle G Bernardi G Identification of the gene-richest bands in human prometaphase chromosomes Chromosom Res 1999 7 379 386 10.1023/A:1009220131225 10515213

[B28] Elstner M Andreoli C Klopstock T Meitinger T Prokisch H The mitochondrial proteome database: MitoP2 Methods Enzym 2009 457 3 20 10.1016/S0076-6879(09)05001-0 19426859

[B29] Smith AC Blackshaw JA Robinson AJ MitoMiner: a data warehouse for mitochondrial proteomics data Nucleic Acids Res 2012 40 D1160 D1167 doi:10.1093/nar/gkr1101 10.1093/nar/gkr1101 22121219 PMC3245170

[B30] Martin W Müller M The hydrogen hypothesis for the first eukaryote Nature 1998 392 37 41 doi:10.1038/32096 10.1038/32096 9510246

[B31] Brown JR Ancient horizontal gene transfer Nat Rev Genet 2003 4 121 132 doi:10.1038/nrg1000 10.1038/nrg1000 12560809

[B32] Mans B Anantharaman V Aravind L Comparative genomics, evolution and origins of the nuclear envelope and nuclear pore complex Cell cycle 2004 3 1612 1637 10.4161/cc.3.12.1345 15611647

[B33] Zimmer C On the origin of eukaryotes Science 2009 325 666 667 10.1126/science.325_666 19661396

[B34] Keller A Vosshall LB Better smelling through genetics: mammalian odor perception Curr Opin Neurobiol 2008 18 364 369 doi:10.1016/j.conb.2008.09.020 10.1016/j.conb.2008.09.020 18938244 PMC2590501

[B35] Cabral A Sayin A De Winter S Fischer DF Pavel S Backendorf C SPRR4, a novel cornified envelope precursor: UV-dependent epidermal expression and selective incorporation into fragile envelopes J Cell Sci 2001 114 3837 3843 11719550 10.1242/jcs.114.21.3837

[B36] McCright B Lozier J Gridley T A mouse model of Alagille syndrome: Notch2 as a genetic modifier of Jag1 haploinsufficiency Development 2002 129 1075 1082 11861489 10.1242/dev.129.4.1075

[B37] Wang D Zhi X Zhang S Jiang M Liu P Han X Li J Chen Z Wang C The bone morphogenetic protein antagonist Gremlin is overexpressed in human malignant mesothelioma Oncol Rep 2012 27 58 64 doi:10.3892/or.2011.1463 21935575 10.3892/or.2011.1463

[B38] Wang K Holterman A-X Pathophysiologic role of hepatocyte nuclear factor 6 Cell Signal 2012 24 9 16 doi:10.1016/j.cellsig.2011.08.009 10.1016/j.cellsig.2011.08.009 21893194

[B39] Chen J-KJ Harrisa RC Angiotensin II induces epithelial-to-mesenchymal transition in renal epithelial cells through reactive oxygen species/Src/caveolin-mediated activation of an epidermal growth factor receptor-extracellular signal-regulated kinase signaling pathway Mol Biol Evol 2012 Published doi:10.1128/MCB.06410-11 10.1128/MCB.06410-11 PMC3295195 22215616

[B40] Wilson JX The renin-angiotensin system in nonmammalian vertebrates Endocr Rev 1984 5 45 61 10.1210/edrv-5-1-45 6368215

[B41] Wagner GP Amemiya C Ruddle F Hox cluster duplications and the opportunity for evolutionary novelties Proc Natl Acad Sci USA 2003 100 14603 14606 doi:10.1073pnas.2536656100 10.1073/pnas.2536656100 14638945 PMC299744

[B42] Hoegg S Meyer A Hox clusters as models for vertebrate genome evolution Trends Genet 2005 21 421 4 doi:10.1016/j.tig.2005.06.004 15967537 10.1016/j.tig.2005.06.004

[B43] Lehmann EL Statistical Methods Based on Ranks 2006 Springer

[B44] Bernardi G The vertebrate genome: isochores and evolution Mol Biol Evol 1993 10 186 204 8450755 10.1093/oxfordjournals.molbev.a039994

[B45] Ohno S Evolution by Gene Duplication 1970 Heidelberg: Springer-Verlag

[B46] Kasahara M The 2R hypothesis: an update Curr Opin Immunol 2007 19 547 52 doi:10.1016/j.coi.2007.07.009 10.1016/j.coi.2007.07.009 17707623

[B47] Tschopp P Fraudeau N Béna F Duboule D Reshuffling genomic landscapes to study the regulatory evolution of Hox gene clusters Proc Natl Acad Sci USA 2011 108 10632 7 doi:10.1073/pnas.1102985108 10.1073/pnas.1102985108 21670281 PMC3127922

[B48] Mezler M Fleischer J Breer H Characteristic features and ligand specificity of the two olfactory receptor classes from Xenopus laevis J Exp Biol 2001 204 2987 2997 11551987 10.1242/jeb.204.17.2987

[B49] Breer H Olfactory receptors: molecular basis for recognition and discrimination of odors Anal Bioanal Chem 2003 337 427 33 doi:10.1007/s00216-003-2113-9 12898108 10.1007/s00216-003-2113-9

[B50] Niimura Y Evolutionary dynamics of olfactory receptor genes in chordates: interaction between environments and genomic contents Hum Genomics 2009 4 107 18 10.1186/1479-7364-4-2-107 20038498 PMC3525206

[B51] Jackson B Tilli CMLJ Hardman MJ Avilion AA MacLeod MC Ashcroft GS Byrne C Late cornified envelope family in differentiating epithelia–response to calcium and ultraviolet irradiation J Invest Dermatol 2005 124 1062 70 doi:10.1111/j.0022-202X.2005.23699.x 10.1111/j.0022-202X.2005.23699.x 15854049

[B52] Magdalini K Marcel H Daniel H The human epidermal differentiation complex: cornified envelope precursors, S100 proteins and the “fused genes” family Exp Dermatol 2012 21 9 643 9 doi: 10.1111/j.1600-0625.2012.01472.x 22507538 10.1111/j.1600-0625.2012.01472.x

[B53] Moon S Cho S Kim H Organization and evolution of mitochondrial gene clusters in human Genomics 2008 92 85 93 doi:10.1016/j.ygeno.2008.01.004 10.1016/j.ygeno.2008.01.004 18559289

[B54] Woischnik M Moraes CT Pattern of organization of human mitochondrial pseudogenes in the nuclear genome Genome Res 2002 12 885 893 doi:10.1101/gr.227202 10.1101/gr.227202 12045142 PMC1383742

[B55] Dehal P Boore JL Two rounds of whole genome duplication in the ancestral vertebrate PLoS Biol 2005 3 e314 doi:10.1101/gr.227202 10.1371/journal.pbio.0030314 16128622 PMC1197285

[B56] Putnam NH Butts T Ferrier DEK Furlong RF Hellsten U Kawashima T Robinson-Rechavi M Shoguchi E Terry A Yu J-K Benito-Gutiérrez EL Dubchak I Garcia-Fernàndez J Gibson-Brown JJ Grigoriev IV Horton AC de Jong PJ Jurka J Kapitonov VV Kohara Y Kuroki Y Lindquist E Lucas S Osoegawa K Pennacchio LA Salamov AA Satou Y Sauka-Spengler T Schmutz J Shin-I T The amphioxus genome and the evolution of the chordate karyotype Nature 2008 453 1064 71 doi:10.1038/nature06967 10.1038/nature06967 18563158

[B57] Larhammar D Lundin L-G Hallböök F The human Hox-bearing chromosome regions did arise by block or chromosome (or even genome) duplications Genome Res 2002 12 1910 20 doi:10.1101/gr.445702 10.1101/gr.445702 12466295 PMC187569

[B58] King MC Wilson A Evolution at two levels in humans and chimpanzees Science 1975 188 107 116 10.1126/science.1090005 1090005

[B59] Carroll SB Evolution at two levels: on genes and form PLoS Biol 2005 3 1159 66 doi:10.1371/journal.pbio.0030245 10.1371/journal.pbio.0030245 PMC1174822 16000021

[B60] Sabherwal N Bangs F Röth R Weiss B Jantz K Tiecke E Hinkel GK Spaich C Hauffa BP van der Kamp H Kapeller J Tickle C Rappold G Long-range conserved non-coding SHOX sequences regulate expression in developing chicken limb and are associated with short stature phenotypes in human patients Hum Mol Gen 2007 16 210 22 doi:10.1093/hmg/ddl470 17200153 10.1093/hmg/ddl470

[B61] Durand C Bangs F Signolet J Decker E Tickle C Rappold G Enhancer elements upstream of the SHOX gene are active in the developing limb Eur J Hum Genet 2010 18 527 32 doi:10.1038/ejhg.2009.216 10.1038/ejhg.2009.216 19997128 PMC2987325

[B62] Ji Y Zhang Z Hu Y The repertoire of G-protein-coupled receptors in Xenopus tropicalis BMC Genomics 2009 10 263 10.1186/1471-2164-10-263 19508718 PMC2709155

[B63] Aloni R Olender T Lancet D Ancient genomic architecture for mammalian olfactory receptor clusters Genome Biol 2006 7 R88 doi:10.1186/gb-2006-7-10-r88 10.1186/gb-2006-7-10-r88 17010214 PMC1794568

[B64] Bergboer JGM Tjabringa GS Kamsteeg M Van Vlijmen-Willems IMJJ Rodijk-Olthuis D Jansen PAM Thuret J-Y Narita M Ishida-Yamamoto A Zeeuwen PLJM Schalkwijk J Psoriasis risk genes of the late cornified envelope-3 group are distinctly expressed compared with genes of other LCE groups Am J Pathol 2011 178 1470 1477 10.1016/j.ajpath.2010.12.017 21435436 PMC3078472

[B65] Wu D-D Irwin DM Zhang Y-P Molecular evolution of the keratin associated protein gene family in mammals, role in the evolution of mammalian hair BMC Evol Biol 2008 8 241 doi:10.1186/1471-2148-8-241 10.1186/1471-2148-8-241 18721477 PMC2528016

[B66] Malik HS Henikoff S Phylogenomics of the nucleosome Nat Struct Biol 2003 10 882 91 doi:10.1038/nsb996 10.1038/nsb996 14583738

[B67] Hudson NJ Gu Q Nagaraj SH Ding Y-S Dalrymple BP Reverter A Eukaryotic evolutionary transitions are associated with extreme codon bias in functionally-related proteins PLoS One 2011 6 e25457 doi:10.1371/journal.pone.0025457 10.1371/journal.pone.0025457 21966531 PMC3179510

[B68] Cohen N Dagan T Stone L Graur D GC composition of the human genome: in search of isochores Mol Biol Evol 2005 22 1260 72 doi:10.1093/molbev/msi115 10.1093/molbev/msi115 15728737

[B69] Costantini M Clay O Auletta F Bernardi G An isochore map of human chromosomes Genome Res 2006 16 536 41 doi:10.1101/gr.4910606 10.1101/gr.4910606 16597586 PMC1457033

[B70] Huang DW Sherman BT Lempicki RA Bioinformatics enrichment tools: paths toward the comprehensive functional analysis of large gene lists Nucleic Acids Res 2009 37 1 13 doi:10.1093/nar/gkn923 10.1093/nar/gkn923 19033363 PMC2615629

[B71] Benjamini Y Hochberg Y Controlling the false discovery rate: a practical and powerful approach to multiple testing J R Stat Soc B 1995 57 289 300

