# Supplementary material for: Implications of human genome structural heterogeneity: functionally related genes tend to reside in organizationally similar genomic regions
Source: BMC Genomics. 2014 Mar 31;15:252. doi: 10.1186/1471-2164-15-252 (PMC4234528; doi:10.1186/1471-2164-15-252)
Supplement: Additional file 1 — An example of table, presenting GO terms, proved to be significantly enriched in corresponding OP group, Benjamini p-values of this enrichments and genes, related to each GO term. [file 1471-2164-15-252-S1.docx]

The Organizational Pattern (OP) groups, GO terms enriched in these groups with corresponding Benjamini p-values of the GO term enrichments, and genes providing this enrichment are shown in three separate Excel files (for “L2”, “H1” and “H2” GC groups, correspondingly). The result of each OP group is presented on a separate spreadsheet of corresponding Excel file (only OP groups with significant enrichments are listed). GO terms and p-values are shown in first and second rows, while gene names and listed in the first row. In the case of gene related to GO term corresponding cell is marked by “v” (fig.1).

GC group names, OP group names and number of significantly enriched GO terms presented in Additional table 1.


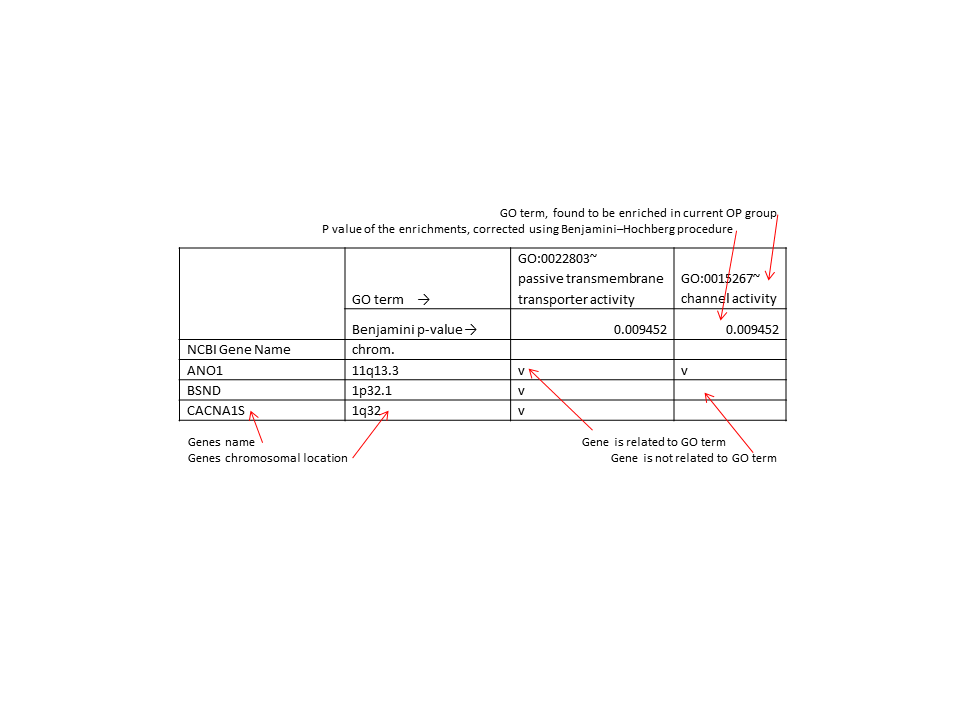


**Additional Fig. 1.** An example of table, presenting GO terms, proved to be significantly enriched in corresponding OP group, Benjamini p-values of this enrichments and genes, related to each GO term.

**Additional table 1.** Organizational pattern clusters names and number of GO terms enriched in these clusters

| GC group name | OP groups name | Number of significantly enriched GO terms |
| --- | --- | --- |
| L2 | a | 16 |
|  | b | 5 |
|  | c | - |
|  | d | - |
|  | e | - |
|  | f | - |
|  | g | 3 |
|  | h | 5 |
|  | i | - |
| H1 | a | - |
|  | b | - |
|  | c | - |
|  | d | - |
|  | e | 10 |
|  | f | - |
|  | g | - |
|  | h | 5 |
|  | i | 7 |
|  | j | 4 |
|  | k | - |
|  | l | - |
|  | m | 4 |
|  | n | - |
|  | o | - |
|  | p | - |
| H2 | a | 24 |
|  | b | 2 |
|  | c | - |
|  | d | - |
|  | e | 5 |
|  | f | 1 |
|  | g | - |
